# Supplementary material for: The effect of tart cherry juice compared to a sports drink on cycling exercise performance, substrate metabolism, and recovery
Source: PLoS One. 2024 Aug 14;19(8):e0307263. doi: 10.1371/journal.pone.0307263 (PMC11324131; doi:10.1371/journal.pone.0307263)
Supplement: Attachment — Submitted filename: Responses to reviewer comments2.docx [file pone.0307263.s002.docx]

**Responses to reviewer comments**

Reviewer #1: The authors provided detailed responses and edited the manuscript with more references regarding tart cherry juice. They also clarified methods that were lacking and data that was missing from the results. I selected minor revisions, because I would like the authors to confirm that power output was maintained during the 90 minutes. I think the results would benefit from a figure that shows the power output during the 90min cycling test.

**Response**: Power output was maintained throughout the test by the researcher watching closely that the participants maintained the required pedaling rate. We have added a statement on this to the manuscript (lines 164-166). We do not have the data for a figure showing the power output during the test.

One minor comment is the first panel of figure one y-axis is labeled "pre-exercise glucose" and the x axis is labeled with times post exercise.

**Response**: The “post” indicators on the x-axis of the graph refer to post-drink consumption and not post-exercise. We have clarified this in the legend for the figure (line 270) .

Also, it doesn't appear that post exercise blood pressure is discussed in the results section.

**Response:** The blood pressure results are mentioned on line in the results section (line 275).

The authors added results regarding excluded participant data that was not in the original version, the participant numbers should be included in the figure/table legends.

**Response**: We have added the participant numbers to the figure legends and blood pressure table, and in the text of the results section as suggested.
